# Pediatric non-urgent emergency department visits and prior care-seeking at primary care

**DOI:** 10.1186/s12913-021-06480-7

**Published:** 2021-05-17

**Authors:** Nithin Ravi, Katherine M. Gitz, Danielle R. Burton, Kristin N. Ray

**Affiliations:** 1 UPMC Children’s Hospital of Pittsburgh, 4401 Penn Avenue,AOB – Suite 5400, Pittsburgh, PA 15224 USA; 2 Children’s Hospital of Los Angeles Medical Group, Los Angeles, USA; 3 Department of Pediatrics, University of Pittsburgh School of Medicine, Pittsburgh, PA USA

**Keywords:** Non-urgent, Nonurgent, Low acuity, Pediatric emergency department, Primary care, Access

## Abstract

**Background:**

We aimed to examine how caregiver perceptions of primary care affects care-seeking prior to pediatric non-urgent ED visits.

**Methods:**

We performed a cross-sectional survey of caregivers of children presenting to a pediatric ED during weekday business hours and triaged as low acuity. We first compared caregiver sociodemographic characteristics, perceptions of primary care, and stated preference in care sites (ED vs PCP) for caregivers who had sought care from their child’s PCP office versus had not sought care from their child’s PCP office prior to their ED visit. We then examined odds of having sought care from their PCP office prior to their ED visit using multivariable logistic regression models sequentially including caregiver primary care perceptions and stated care site preferences along with caregiver sociodemographic characteristics.

**Results:**

Of 140 respondents, 64 (46%) sought care from their child’s PCP office prior to presenting to the ED. In unadjusted analysis, children insured by Medicaid or CHIP, caregivers identifying as Black, and caregivers with lower educational attainment were less likely to have sought PCP care before presenting to the ED (*p* < 0.005, each). Caregivers who had sought PCP care were more likely to prefer their PCP relative to the ED in terms of ease of travel, cost, and wait times (*p* < 0.001, all). When including these stated preferences in a multivariable model, child insurance, caregiver race, and caregiver education were no longer significantly associated with odds of having sought PCP care prior to their ED visit.

**Conclusions:**

Differential access to primary care may underlie observed demographic differences in non-urgent pediatric ED utilization.

## Background

In 2016 the Centers for Disease Control and Prevention estimated that 40% of ED visits for children 4 years old and under received initial triage levels of semi- or nonurgent [1]. These ED visits are often a target for interventions, with the underlying goal of redirecting some of these visits to other sites of care, such as the child’s primary care office [2–4]. Several studies have described associations between non-urgent ED use and child or family sociodemographic characteristics, such as age, race, income, insurance, and social support [5–9]. For example, children with Medicaid coverage are twice as likely as privately insured children to visit the ED, and their ED visits are less likely to be assessed as urgent or emergent [10]. Race has been inconsistently associated with non-urgent ED visits, with a recent systematic review noting that four of nine studies found an association between Black race and non-urgent ED visits, while the remaining identified studies found no association [5]. Lower parental educational attainment and lower health literacy have also been associated with increased nonurgent ED visits [11, 12]. In addition to these sociodemographic factors, many dimensions of primary care access have been associated with non-urgent ED visit rates, including geographic access and perceived quality of care [13–21].

In a recent systematic review, these sets of factors were synthesized into one model that highlighted potential causal pathway factors (e.g., perceptions of access, cost, convenience, quality, advice/referral from PCP) that prompt a patient’s decisions in whether to seek care and where to seek care (PCPs office or the ED) [5]. Unlike the previous studies mentioned, this model distinguished these causal pathway factors from associated sociodemographic factors (e.g., race, insurance) which the authors posited are likely associated with non-urgent ED visits only through their association with potential causal pathway factors [5].

Among the potential causal factors in this pathway, advice or referral from primary care warrants particular attention. After all, children referred to the ED by their primary care provider (PCP) have followed a different pathway to care than children whose families self-referred, and understanding their different perceptions of care and motives may guide different interventions for each group. In prior studies, between one-third and one-half of non-urgent pediatric ED visits and up to 80% of all pediatric ED visits were preceded by contact or attempted contact with the child’s primary care office [14, 22–24]. How other potentially causal factors (e.g., perceptions of primary care access, costs, quality) influence the decision to attempt PCP contact prior to an ED visit are not known, but improved understanding of these heterogeneous routes to non-urgent ED visits may improve understanding of underlying sociodemographic variation in non-urgent ED visits as well.

Thus, to guide future interventions to reduce non-urgent ED visits, we sought to examine patterns of PCP contact prior to non-urgent ED visits through a survey administered to caregivers of children presenting with low acuity ED visits during hours when their PCP would be expected to be open. We examined prevalence of contact with their child’s PCPs office prior to the child’s non-urgent ED visit, and compared caregiver perceptions of their child’s PCP office and trust in their PCP as primary exposures in care seeking decisions. Our secondary analysis examined caregivers’ relative assessment of primary care versus emergency care among those who did and did not seek care from their PCP’s office prior to their non-urgent ED visit. We examined whether these perceptions and care preferences explained the variation in sociodemographic composition of the two groups.

## Methods

We performed a cross-sectional survey of caregivers accompanying children to a pediatric ED during weekday business hours and triaged as low acuity. This study focuses on the association between care-seeking prior to presenting to the ED and respondent sociodemographic characteristics, primary care perceptions, and relative assessment of sites of care (ED vs PCP); a separate analysis examined non-response to health-related social need questions within the survey [25]. This study received ethical approval from our institution’s Institutional Review Board, who approved a waiver to document informed consent due to the study involving no more than minimal risk of harm.

### Survey design

We developed a survey which included items on sociodemographic characteristics, primary care perceptions, health care utilization, and care decision-making; the survey items developed for this study are available as a supplemental file of a prior manuscript [25]. To understand the path that patients took prior to presenting with a non-urgent concern, we asked whether caregivers had attempted to contact their PCP prior to their ED visit. To assess perceptions of their primary care provider, we used the validated, 23-item Parents Perception of Primary Care (P3C) questionnaire [26]. The 23-item P3C yields a total score and subscale scores for continuity, access, contextual knowledge, communication, comprehensiveness, and coordination. To assess trust in primary care, we used the validated 5-item Trust in Provider Scale [27]. The Cronbach’s coefficient alpha for the P3C scale is 0.95, and the Cronbach’s coefficient alpha for the trust in physician/provider scale is 0.87 [26, 27]. To determine relative assessment of alternative sites of care, caregivers were asked to identify whether their PCP or the ED was better across dimensions identified as potentially relevant in prior models of access to healthcare in general [28, 29] and of non-urgent ED visits specifically [5], including wait times, travel time, quality of care, and out-of-pocket costs. The survey also asked respondents about their age, race, ethnicity, educational attainment, household members, and child insurer as well as likely care seeking in future clinical scenarios.

### Survey administration

Participants were recruited by trained research assistants during visits to the UPMC Children’s Hospital of Pittsburgh pediatric emergency department. Recruitment occurred on random weekdays over a 5-month period (February 2019–June 2019), with all caregivers during these days approached if their child was ≤5 years old, presented to the ED on a weekday between the hours of 8 am – 5 pm, and received initial triage scores indicating low acuity (level 4 or 5 based on the Emergency Severity Index) [30]. We focused on the youngest children because of the higher rates of non-urgent ED visits in this population. We recruited during weekday business hours since a primary care visit could have likely been an alternative option for care for visits during those hours. Caregivers unable to consent in English were excluded. If multiple caregivers were present, we asked that they choose one caregiver to complete the entirety of the survey. Research assistants identified and approached potentially eligible participants during their child’s ED visit and obtained verbal consent. After obtaining and electronically documenting informed consent, the research assistant provided an iPad that contained the self-administered electronic survey that caregivers completed alone, while research assistants waited outside the room in the event of additional questions. After the survey, a resource guide was provided to all families that addressed resources for obtaining health insurance, locating a primary care physician, and receiving immunizations. Recruitment continued until a goal of 150 participants, which we estimated would yield adequate power (> 0.80) to detect a 12-point difference in the P3C score and a 2-point difference in the Trust in Provider scale in samples across a range of group allocation ratios (1:1 to 1.5:1) [26, 27]. In prior studies, groups with meaningful differences in access to care had P3C scores that varied by 13–16 points [26].

### Preparing analytic sample

Individuals who started but did not complete the survey, did not respond to any demographic variables, or who did not respond to the item inquiring about care-seeking prior to their child’s ED visit were excluded from the analytic sample.

#### Care-seeking at primary care before ED visit

Respondents who indicated that they did not try to contact their doctor’s office before coming to the ED were coded as not attempting contact with their PCP. All other responses were coded as attempting contact, with these responses including “Yes, I had talked to my doctor’s office”, “Yes, I was seen in my doctor’s office”, and “No, I tried to call my doctor’s office but did not get a response.” For those indicating that they either talked with or were seen at their doctor’s office prior to coming to the ED, an additional item asked whether the pediatrician recommended they come to the ED.

#### Primary care perceptions, trust, and preferred site

Responses to the Parent Perceptions of Primary Care survey items were coded according to published methods, resulting in a continuous variable from 0 to 100, with 100 being optimal perception [26]. Trust in Provider was also coded according to published methods, yielding a continuous score from 5 to 25, with 25 being highest trust [27]. For questions assessing relative preference in care between the ED and their PCP based on specific criteria (wait time, travel time, quality of care, and out-of-pocket costs), those indicating that both options were equal and those not responding were grouped together as a “neutral” response, as compared to those stating a preference for either the ED or primary care.

#### Respondent sociodemographic characteristics

Due to small counts for specific levels of some categories, we recoded race, educational attainment, and child insurance for analysis. For race, we grouped individuals identifying as Asian-American (*n* = 3) along with those identifying as “other” race (*n* = 9), resulting in 4 categories for race: white, Black/African American, other, and missing. For educational attainment, individuals reporting not having completed high school (*n* = 7) were grouped along with those reporting completing high school or a General Educational Development (GED) (*n* = 36). For child insurance status, the small number of individuals reporting no insurance (*n* = 2) were combined with those who selected “other” (*n* = 10) and who did not respond (n = 1). Responses were included as observations for all variables in our final analysis.

### Statistical analyses

We used descriptive statistics to describe the demographic characteristics of the sample and reported care-seeking at PCPs office prior to presenting to the ED. We compared caregivers who reported that they had and had not sought care from their child’s PCP prior to presenting to the ED by sociodemographic characteristics, using chi-squared tests or Fischer exact tests depending on expected cell size.

Next, we examined the association between perceptions of primary care, trust in PCP, and relative assessments of primary care versus ED care and care-seeking at PCPs office before ED visit using Kruskal-Wallis tests for continuous data and chi-squared tests for categorical data.

Finally, we examined whether perception of their PCP and relative preferences in care site mediated the variation in sociodemographic composition of caregivers who had and had not sought care prior to their non-urgent ED visit. For this analysis, we tested these relationships with three sequential logistic regression models, using likelihood ratio tests to compare models. For each, prior PCP care-seeking was the binary dependent variable. In the first model, we included as independent variables the sociodemographic variables found to be significant (*p* < 0.05) in bivariate analysis (caregiver race, educational attainment, and child insurance). In the second model, we added as additional independent variables the parent perception of primary care subscores found to be significant in bivariate analysis (access, contextual knowledge, and care coordination subscores) to the sociodemographic variables. In the third model, we added significant relative assessment responses (travel choice, wait times, and cost of ED vs PCP) instead of primary care perception subscores as an alternative set of factors with the potential to mediate sociodemographic associations with care-seeking. Study data were collected using Qualtrics (Qualtrics, Provo, Utah) and analyzed using Stata IC 15 (StataCorp, College Station, TX). Significance testing was performed using an alpha level of 0.05.

## Results

One hundred sixty nine caregivers were approached, and 157 of these agreed to participate. After excluding those with missingness of key variables, 140 are included in the final analytic sample. Respondents were primarily between the ages of 25–44 years old, with 51% identifying as white and 29% as Black or African American (Table 1). Most respondents reported attending the ED with a child insured by Medicaid or CHIP (62%), and 40 % had completed college or post-college education.

**Table 1:** Respondent sociodemographic characteristics and care-seeking from their child’s PCP prior to their child’s ED visit

	All Respondents	Sought care from PCP before ED		*P*-value
		Yes	No	
	n (%)	n (row %)	n (row %)	
**Respondents**	**140**	**64**	**76**	
**Caregiver Age**				0.21
< 25 Years	32 (23%)	12 (38%)	20 (62%)	
≥ 25 Years	106 (76%)	50 (47%)	56 (53%)	
Missing	2 (1%)	2 (100%)	0 (0%)	
**Caregiver Race**				<.001*
White	71 (51%)	44 (62%)	27 (38%)	
Black or African-American	41 (29%)	10 (24%)	31 (76%)	
Other	12 (9%)	6 (50%)	6 (50%)	
Prefer Not to Say/Missing	16 (11%)	4 (25%)	12 (75%)	
**Caregiver Ethnicity**				0.11
Hispanic or Latino	6 (4%)	4 (67%)	2 (33%)	
Not Hispanic or Latino	112 (80%)	54 (48%)	58 (52%)	
Prefer Not to Say/Missing	22 (16%)	6 (27%)	16 (73%)	
**Caregiver Educational Level**				.001*
High School/GED or Less	43 (31%)	10 (23%)	33 (77%)	
Some College	39 (28%)	18 (46%)	21 (54%)	
Completed College/Post-college	56 (40%)	34 (61%)	22 (39%)	
Missing	2 (1%)	2 (100%)	0 (0%)	
**Child Insurance**				.004*
Commercial/Employer Based	40 (29%)	27 (68%)	13 (32%)	
Medicaid or CHIP	87 (62%)	31 (36%)	56 (64%)	
No insurance/Other/Missing	13 (9%)	6 (46%)	7 (54%)	
**Child Age**				0.25
0–1 Years	25 (18%)	14 (56%)	11 (44%)	
≥ 1 Years	115 (82%)	50 (43%)	65 (57%)	
**Number of Household Children**				0.68
1 Child	43 (31%)	18 (42%)	25 (58%)	
≥ 1 Child	94 (67%)	44 (47%)	50 (53%)	
Missing	3 (2%)	2 (67%)	1 (33%)	

Legend: Unadjusted analysis examining association between sociodemographic characteristics and having sought care from their child’s PCP office prior to their child’s ED visits, using chi-square or Fischer’s exact test. *PCP* Primary care provider, *ED* Emergency department

* = Statistically significant difference (α = .05)

**Fig. 1:**
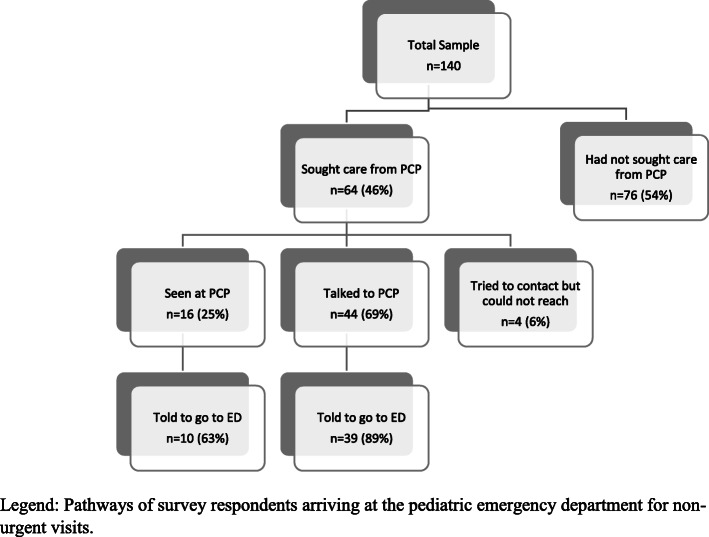
Healthcare Utilization Pathway of Survey Respondents

Of respondents, 46% (64/140) reported that they had sought care from their child’s PCP office prior to presenting to the ED, while the remaining 54% reported they had not sought care from their child’s PCP office (Fig. 1). Of those who had sought care from their child’s PCP office, 25% (16/64) reported their child had a visit with their PCP, 69% (44/64) reported they talked with their child’s PCP office via telephone, and 6% (4/64) reported they attempted to contact the office but did not reach them. Of those who spoke with or saw their PCP’s office, 82% (49/60) were told to present to the emergency department, meaning 35% of the entire sample (49/140) had been instructed by their PCP’s office to present to the ED.

Caregivers reporting that they sought care from their child’s PCP office prior to their child’s ED visits differed significantly by child insurance status, caregiver race, and caregiver educational attainment (Table 1). Among caregivers of children triaged as low acuity in the ED, only 36% of those insured by Medicaid or CHIP had sought PCP care before presenting to the ED, compared to 68% of those insured by commercial insurance (*p* = 0.004). 62% of white caregivers presenting to the ED for a low acuity concern had sought PCP care prior to presenting to the ED compared to 24% of caregivers who identified as Black (*p* < 0.001). 77% of caregivers with a high school education/GED or less had not sought PCP care prior to presenting to the ED compared to 39% of those reporting at least a college education (*p* = 0.001).

Those who sought care from their PCP’s office prior to coming to the ED were significantly more likely to report higher median perception of primary care, with total score of 86 (IQR 71–92) for those who had sought PCP care and 76 (IQR 60–90) for those who had not sought PCP care (*p* = 0.01; Table 2). Significant variation was identified for three specific subscores: access, contextual knowledge, and coordination. For example, median access subscore was higher for those who had sought PCP care than those who had not (88 vs. 75, *p* < 0.001). Trust in provider did not significantly differ for caregivers who had and had not sought care from their child’s PCP office (*p* = 0.12).

**Table 2:** Primary Care Contexts Associated with Caregivers Reporting Having Sought Care from PCP prior to ED visit

	Sought care from PCP before ED		*P*-Value
	Yes	No	
	Median (IQR)	Median (IQR)	
**Parent Perceptions of Primary Care**			
Respondents, n	64	76	
Total Score	86 (71–92)	76 (60–90)	0.01*
Subscores			
Continuity	60 (35–80)	60 (40–80)	0.48
Access	88 (69–100)	75 (53–84)	<.001*
Contextual Knowledge	94 (81–100)	81 (56–100)	.02*
Communication	100 (81–100)	94 (75–100)	.09
Comprehensiveness	90 (70–100)	85 (65–100)	.26
Coordination	85 (65–100)	75 (38–97)	.02*
**Trust in Primary Care Provider**			
Respondents, n	62	74	
Trust in Provider Score	22 (20–25)	21 (18–24)	0.12

Legend: Parent perceptions of primary care and trust in primary care provider for respondents who did and did not report attempting to contact their PCP's office prior to their child’s ED visit. Unadjusted analysis using Kruskal-Wallis tests. *PCP* Primary care provider, *ED* Emergency department, *IQR* Interquartile range. Four respondents did not provide answers to the trust in provider items.

*Statistically significant difference (α = .05)

Caregiver’ perceptions of travel time, wait time, and costs at their child’s PCP versus the ED were significantly associated with prior PCP care-seeking (Table 3). Among caregivers of children triaged as low acuity in the ED, 69% of those who reported travel time to their PCP was better than to the ED had sought prior PCP care compared to 18% of those who reported travel time to the ED was better (*p* < .001). Among respondents who reported wait time at their PCP was better than in the ED, 67% had sought prior PCP care compared to only 10% of those reporting ED wait time was better (*p* < .001). Assessment of quality of care between the two sites was not associated with prior care-seeking (*p* = 0.49).

**Table 3:** Relative assessment of care choice between PCPs office vs ED and prior PCP care-seeking

	Sought care from PCP before ED		*p-*value
	Yes	No	
	n (row %)	n (row %)	
**Respondents**	**64**	**76**	
Travel Preference			<.001*
ED Better	3 (18%)	14 (82%)	
Neutral	19 (31%)	43 (69%)	
PCP Better	42 (69%)	19 (31%)	
Wait Time Preference			<.001*
ED Better	2 (10%)	19 (90%)	
Neutral	23 (38%)	38 (62%)	
PCP Better	39 (67%)	19 (33%)	
Better Care Preference			
ED Better	14 (39%)	22 (61%)	0.49
Neutral	42 (47%)	48 (53%)	
PCP Better	8 (57%)	6 (43%)	
Payment Preference			
ED Better	0	0	.002*
Neutral	41 (38%)	66 (62%)	
PCP Better	23 (70%)	10 (30%)	

Legend: Relative assessment of care settings (PCP vs. ED) and respondent report of prior PCP care-seeking. Unadjusted analysis chi-square analysis or fischer’s exact text

*Statistically significant difference (α = .05)

In the first multivariable model including only sociodemographic variables (child insurance, parent race, and parent education; Table 4**Model A**), odds of having sought care from their child’s PCP office prior to their non-urgent ED visit were significantly lower for parents who had not entered college (OR 0.32, 95% CI 0.11–0.90) compared to those who had completed college and for parents identifying as Black or African American (OR 0.29, 95% CI 0.11–0.74) compared to parents identifying as white. When also including significant perception of primary care subscales (access, contextual knowledge, coordination, Table 4**Model B**), the model was not significantly improved (likelihood ratio (LR) test, *p* = 0.18), and sociodemographic associations maintained significance with similar magnitude and direction. When instead including significant relative assessments of site of care (travel, wait times, and cost estimations), the model was significantly improved (LR test *p* < 0.001). In this final model (Table 4**, Model C**), estimates of PCP wait time and travel time being better than ED wait time and travel time were significantly associated with odds of having sought care from child’s PCP office prior to their non-urgent ED visit (Travel: PCP better option, OR 3.46, 95% CI 1.34–8.92 compared to neutral response reference group; Wait time: PCP better option, OR 2.86, 95% CI 1.11–7.34 compared to neutral response reference group). In this final model accounting for parents’ relative assessments of care sites, odds ratios associated with Black parent race and lower educational attainment were reduced by 33 and 6%, respectively, and were no longer significantly associated with odds of having sought care from their child’s PCP office prior to their non-urgent ED visit.

**Table 4:** Adjusted Odds of Having Sought Care from PCP before ED

Sociodemographic Factors	Model A^a^	Model B	Model C
**N**	**140**	**140**	**140**
**Likelihood Ratio Test (*P-*Value)**	Reference	.18	<.001*
	**OR (95% CI)**	**OR (95% CI)**	
**Respondent Demographics**			
**Insurance Status**			
Commercial/Employer Based	Reference	Reference	Reference
Medicaid or CHIP	.56 (.21, 1.48)	.66 (.24, 1.84)	.44 (.09, 2.15)
No insurance/Other/Missing	.77 (.18, 3.34)	1.04 (.22, 4.94)	.64 (.09, 4.60)
**Race**			
White	Reference	Reference	Reference
African-American	.29 (.11, .74)*	.32 (.11, .88)*	.43 (.14, 1.31)
Asian/Native American/Other	.99 (.26, 3.76)	1.07 (.28, 4.13)	2.76 (.48, 15.73)
Prefer Not to Say/Missing	.19 (.05, .80)*	.23 (.05, 1.01)	.25 (.05, 1.27)
**Education**			
Completed College/Post-college	Reference	Reference	Reference
Some College	.76 (.29, 1.9)	.73 (.27, 1.98)	.50 (.16, 1.59)
High School/GED or Less	.32 (.11, .90)*	.30 (.10, .88)*	.34 (.10, 1.08)
**Respondent PCP Perception**			
**Subscales**			
Access		1.01 (.98, 1.03)	
Contextual Knowledge		1.02 (.99, 1.04)	
Coordination		1.00 (.98, 1.02)	
**Respondent Preferences**			
**Travel Choice**			
ED Better			.64 (.13, 3.19)
Neutral			Reference
PCP Better			3.46 (1.34, 8.92)*
**Wait Time Choice**			
ED Better			0.19 (.03, 1.13)
Neutral			Reference
PCP Better			2.86 (1.11, 7.34)*
**Payment Choice**			
ED Better			(No observations)
Neutral			Reference
PCP Better			.70 (.14, 3.58)

*Statistically significant difference (α = .05)

Model A: Sought care from PCP prior to ED = Insurance Status + Race + Education

Model B: Sought care from PCP prior to ED = Insurance Status + Race + Education + P3C Access Subscore + P3C Contextual Knowledge Subscore + P3C Coordination of Care Subscore

Model C: Sought care from PCP prior to ED = Insurance Status + Race + Education + Relative Assessment of Travel Time + Relative Assessment of Wait Time + Relative Assessment of Payment/Cost

## Discussion

One of the key findings from our cross-sectional survey of caregivers of children presenting for a non-urgent ED visit is that pediatric patients follow multiple different pathways when they present to the ED for visits that are triaged as low acuity. Consistent with prior estimates [22, 23], we found that nearly half of surveyed caregivers had sought advice from their PCP’s office before presenting to the ED. By identifying differences in caregivers’ perceptions of their primary care provider and relative preferences regarding the care they receive at their PCPs office versus the ED, our results suggest ways that prior primary care and ED experiences may influence decisions in where to seek care. Notably, primary care perceptions associated with seeking PCP care appear primarily related to convenience (access, care coordination), and contextual knowledge as opposed to quality, comprehensiveness, or communication. Our results also suggest that caregivers’ perceptions of their child’s PCP office may be less of a decisional factor than the relative assessment comparing potential time and financial costs at the PCP office versus the ED. This suggests that among the causal factors posited in prior conceptual models [5], further work should investigate the decision to seek advice/referral as a key decision point potentially influenced by other identified causal pathway factors (e.g., cost, convenience, access/availability, beliefs about knowledge and alternatives).

A second key finding is that while caregivers with lower educational attainment and caregivers identifying as Black or African American had lower odds of having sought PCP care before non-urgent ED visit, these associations were no longer significant when caregiver relative assessment of wait time, travel time, and cost were included. This lends support to the prior conceptual models [5, 28] that sociodemographic factors such as race, insurance status, and education are associated factors mediated through other specific causal factors in care seeking decisions (e.g., assessments of relative access, convenience, and cost). We note that these findings should be replicated in larger samples, but also that the significant likelihood ratio test (model C versus model A, *p* < 0.001) indicates that the loss of significance with race and educational attainment occurred in the context of an overall improvement in the model. In the United States, experiences of accessing and receiving care are consistently more difficult for individuals identified as Black or Hispanic as well as by educational background or insurer [31]. For example, studies in primary care and emergency department settings have reported that time spent waiting for care varies by race, education, and insurance [32–34]. Implicit bias and structural racism can also shape experiences of care and geographic access to care [31]. These studies illustrate ways that families in the current US health care delivery systems may have health care experiences (e.g., travel time and wait time) that differ systematically by race. As a result, where a caregiver takes their child for non-urgent care may be driven by differential access to primary care across race, education level, and insurance type rather than these characteristics themselves.

Parents of sick children are seeking to make the best decisions for their children given the information and options they have. Thus, patterns of care-seeking deemed sub-optimal by health care professionals and systems should be viewed as symptoms of an imperfect system, rather than imperfect individual decision-making. With this mindset, analyses of non-urgent ED visits can be used to identify ways to change the system to make it easier for parents to obtain the care their child needs in lower cost settings. One possibility suggested by our data is to address travel time or wait time at primary care relative to the ED. One recent study found that implementing walk-in care within the medical home, which may alter wait time, reduced the proportion of care received in ED or urgent care settings for their patients [35]. Further opportunities to reduce travel time and wait time exist through school and childcare-based clinics and telehealth [36, 37] as well as more mundane interventions such as having adequate staffing and efficient scheduling and rooming workflows [38]. As specific examples, use of telemedicine to deliver school-based asthma care and childcare-based acute care have both reduced emergency department visits [36, 37]. Finally, our data suggest that for nearly half of non-urgent ED visits, parents are already contacting and being referred by their PCPs office. Since most caregivers who were recommended to present to the ED by their PCP office spoke with the PCP office over the telephone, future studies could focus on nurse triage systems at these PCP offices as an important alternative intervention point for reducing non-urgent ED visits. Indeed, prior work has demonstrated that physician involvement before nurse triage referral to the ED may reduce ED referrals by half [39]. Alternative strategies to reduce these PCP-referred low acuity ED visits may instead require primary care-ED partnered investigations of shared referral pathways, primary care resources, direct admission strategies, and access to specialty care [40, 41].

### Limitations

Our study involved a cross-sectional survey of parents presenting to the ED, in which we asked parents to report on prior PCP contact. As a result, our study focuses only on the subset of parents with sick children who arrive at the ED, as opposed to those who contact their PCP and stay home or those who consider presenting to the ED and do not. We recruited caregivers through sequential sampling on random days, and we excluded non-English speakers, which may limit the generalizability of our study results. Additionally, we focused on one specific decision point prior to ED visit. We recognize that other decision points exist (such as the decision to seek online information [42]), but this was beyond the scope of our survey. Additionally, we recognize that triage status does not necessarily correlate with need for testing or procedures in the ED [43]. Indeed, in a separate follow-up survey, we reached 75 of these participants and found that 6 (8%) had been admitted. As a result, we avoid labeling these visits as “unnecessary” and focus instead on their low acuity triage status. Finally, we acknowledge that our sample size was powered for our unadjusted analysis; investigating the multivariable associations in larger samples may allow for confirmation of these findings and examination of the additional potential confounders not available in this analysis.

## Conclusions

In conclusion, among caregivers of children presenting to the ED for non-urgent visits (triaged as low acuity), we found that (1) more positive parent perceptions of the convenience and contextual knowledge of their child’s primary care provider and (2) estimations of shorter travel times and wait times at primary care offices compared to the ED were both associated with increased likelihood of having sought care from the child’s PCP prior to a non-urgent ED visit. Differential primary care access by sociodemographic groups in the US may contribute to previously observed sociodemographic differences in non-urgent ED utilization.

## Data Availability

The datasets generated during and/or analyzed during the current study are available from the corresponding author on reasonable request.

## Abbreviations

ED: Emergency department
PCP: Primary care provider
CHIP: Children’s health insurance program
